# Prevalence, Indications, and Community Perceptions of Caesarean Section Delivery in Ngora District, Eastern Uganda: Mixed Method Study

**DOI:** 10.1155/2020/5036260

**Published:** 2020-07-20

**Authors:** Isaac Waniala, Sandra Nakiseka, Winnie Nambi, Isaac Naminya, Margret Osuban Ajeni, Jacob Iramiot, Rebecca Nekaka, Julius Nteziyaremye

**Affiliations:** ^1^Department of Community and Public Health, Faculty of Health Sciences, Busitema University, Busitema, Uganda; ^2^Department of Microbiology and Immunology, Faculty of Health Sciences, Busitema University, Busitema, Uganda; ^3^Department of Obstetrics and Gynaecology, Faculty of Health Sciences, Busitema University, Busitema, Uganda; ^4^Department of Obstetrics and Gynaecology, Mbale Regional Referral and Teaching Hospital, Mbale, Uganda

## Abstract

**Background:**

Uganda has a high maternal mortality ratio (MMR) of 336/100,000 live births. Caesarean section is fundamental in achieving equity and equality in emergency obstetric care services. Despite it being a lifesaving intervention, it is associated with risks. There has been a surge in caesarean section rates in some areas, yet others remain underserved. Studies have shown that rates exceeding 15% do not improve maternal and neonatal morbidity and mortality. Our study aimed at determining the prevalence, indications, and community perceptions of caesarean section delivery in Eastern Uganda.

**Methods and Materials:**

It was both health facility and commuity based cross-sectional descriptive study in Ngora district, Eastern Uganda. Mixed methods of data collection were employed in which quantitative data were collected by retrospectively reviewing all charts of all the mothers that had delivered at the two comprehensive emergency obstetric care service facilities between April 2018 and March 2019. Qualitative data were collected by focus group discussions till point of saturation. Data were entered into EpiData (version 3.1) and analyzed using SPSS software (version 24). Qualitative data analysis was done by transcribing and translating into English verbatim and then analyzed into themes and subthemes with the help of NVIVO 12.

**Results:**

Of the total 2573 deliveries, 14% (357/2573) were by CS. The major single indications were obstructed labour 17.9%, fetal distress 15.3%, big baby 11.6%, and cephalopelvic disproportion (CPD) 11%. Although appreciated as lifesaving for young mothers, those with diseases and recurrent intrauterine fetal demise, others considered CS a curse, marriage-breaker, misfortune, money-maker and a sign of incompetent health workers, and being for the lazy women and the rich civil servants. The rise was also attributed to intramuscular injections and contraceptive use. Overall, vaginal delivery was the preferred route.

**Conclusion:**

Several misconceptions that could hinder access to CS were found which calls for more counseling and male involvement. Although facility based, the rate is higher than the desired 5–15%. It is higher than the projected increase of 36% by 2021. It highlights the need for male involvement during counseling and consent for CS and concerted efforts to demystify community misconceptions about women that undergo CS. These misconceptions may be a hindrance to access to CS.

## 1. Background

In 2015, at the fall of the Millennium Development Goal (MDG) period, Sustainable Development Goals (SDGs) were ushered in. SDG 3.1 aims to reduce the global maternal mortality ratio (MMR) to less than 70 per 100,000 live births by 2030 and to have no country with MMR above 140. Yet, in 2015, about 303,000 women died of maternal causes. Maternal mortality, emerged the second leading cause of death only bettered by the human immune virus among women aged 15–49 [1].

Although globally the lifetime risk of dying due to maternal death is 1 in 180, Africa's high MMR of 540 per 100,000 live births coupled with high fertility levels translates into a lifetime risk of dying from maternal causes of 1 in 37 [2]. Therefore, achieving equity and equality in availability of emergency obstetric care services including assisted vaginal delivery and safe caesarean section (CS) is exceedingly key in the attainment of SDG 3 [3]. Moreover the population-based caesarean section is used as a process indicator in maternal health to monitor progress [4].

Despite being a lifesaving intervention, caesarean section is not without complications that could lead to maternal, neonate, and infant morbidity and mortality and development of chronic noncommunicable diseases. Its high cost may lead to unnecessary expenditure to the already overburdened and economically hard-hit families, especially in the developing countries [5–7]. The World Health Organization has thus declared that for CS to have an optimal impact, it must be between 5 and 15%. WHO worldwide ecological studies have shown that below a caesarean section rate of 10%, maternal and neonatal mortality decreased when caesarean section rates increased. As caesarean section rates increased above 10% and up to 30%, no effect on mortality rates was observed [8–10]Despite that, there has been a tremendous increase in population-based all-cause CS rates globally ranging from 0.4 to 51%. Worth noting is that a continuous rise in the trend has been observed during the past 30 years [11–15].

This trend has also been reflected in the Ugandan studies. A study done in Uganda in which analysis of Health Management Information System (HMIS) data reports between 2012 and 2016 from 3461 health facilities providing basic, essential obstetric, and emergency obstetric care services revealed an overall CS rate for live births at facilities as 9.9%, increasing from 8.5% in 2012 to 11% in 2016. The overall population-based CS rate was 4.7% which increased from 3.2 to 5.9% over the same period. Significantly, Health Centre IV level facilities (these are health subdistrict centers with a medical doctor and are the first level for comprehensive emergency obstetric services) had the largest annual rate of increase in CS between 2012 and 2016. Overall, Uganda's facility-based CS rate was projected to increase by 36% in 2021, while the population-based CS rate was estimated to double in the same period from the baseline in 2016 [16]. A study in Kabarole, Western Uganda, in 2016, established a caesarean rate of 25% with disparities at low- and high-level facilities with the former having a rate of 6–8% and the latter 18–16%. In this study, factors such as having a previous CS, complications during pregnancy, ANC attendance of 4 or more times, inadequate human resource, inadequate medicines and supplies, and myths and misconceptions about CS affected access and utilization of CS services [17]. This rise in the trend has not come without fears, and some countries like Italy have used it to gauge the quality of services provided; the low rate, being commendable as a marker of high-quality services. This initiative was taken after a five-year experience in Abruzzo, Italy, revealed that a third (18.5million) of the caesarean sections had been performed for nonmedical indications [18].

There are several indications for CS, and these can be medical, obstetric, or both [19]. Worrying to this trend though is the increase in maternal request for CS especially in urban areas, in private facilities, and amongst the more affluent populations [13, 19, 20]. That notwithsatnding, there are mixed perceptions about CS with the majority of women and men expressing preference for vaginal deliveries, although more women are willing to undergo the operation if the husband consented to it. [21–23]. Because of paucity of the literature in Eastern Uganda about the common indications and the community perceptions about CS, we set out to answer these questions.

## 2. Methods and Materials

This was a both a community and health facility based cross-sectional study design with mixed methods (qualitative and quantitative methods). For quantitative data, we did a retrospective review of all the maternity charts of mothers that had delivered between April 2018 and March 2019. This was done at the two major health centers, Ngora health Centre IV and Ngora district Maternity Unit—the two are the only ones that provide comprehensive emergency obstetric care services (CEmOC) in the district. From these charts, we were able to review the demographics, obstetric factors, and the indications for the CS. Quantitative data collected from the charts were double-checked for completeness and later entered into EpiData (version 3.1) and analyzed using SPSS software (version 24). Tables and frequencies were used for data summarization.

The study design for the qualitative part was phenomenological. We held focus group discussions (FGDs) with men and women at different times using the interview guides that were developed with the aid of the Busitema University Community Based Education and Research Services (COBERS) department that is experienced in phenomenological studies (Supplementary Materials (available here)). The research assistants were trained in guiding interviews prior to this exercise. We selected community participants with the aid of the village health team members. The participants in these FGDs were purposively selected targeting those mothers who had undergone caesarean section and men whose wives had undergone caesarean section in a period not exceeding one year prior to the study period. One year was considered not too long for a person to remember the events surrounding the event. Each FGD had 6–12 participants and lasted about 1-2 hours. We held two FDGs for men and three for women. We stopped after reaching the point of saturation. Each focus group discussion had a research assistant who also did the recording of the proceedings. The FGDs led by research assistants were held in the major local language Ateso and recorded, transcribed, and translated into English verbatim. Each transcript was analyzed by two researchers working independently to reduce bias using NVIVO software version 12. Coding was done manually based on the key words and phrases developed from the data. The codes were then grouped together into higher-order headings. Accordingly, on a higher logical level of abstractions codes, subcategories, categories, and themes were formed. The themes were categorized according to the perceptions, indications, risk factors, and advantages relating to the caesarean section. The data were sorted out thematically by clustering material with similar content. At this stage, we used creative and analytical reasoning to determine categories of the meaning.

## 3. Results

Number and mode of delivery of women and their age groups, percentage of caesarean section delivery according to parities of mothers, summary of indications for caesarean section delivery in the Ngora district, characteristics of the focus group discussion participants, generated themes and myths generated from the focus group discussion are provide in Tables 1–5.

### 3.1. The Prevalence of C-Section and Indications for Ngora District

A total of 2573 deliveries were realized. Out of the 2573 mothers who delivered in that period, 24% (618/2573) were teenagers (15–19 years), while 36% (926/2573) were young mothers (20–24 years).

Vaginal deliveries were 86% (2216/2573), while 14% (357/2573) were CS deliveries. Significantly, teenagers contributed 27.2% (97/357) of the total. The percentage of caesarean section deliveries ranged from as low as 7% in April 2018 to as high as 27% in November (Table 1). Of the 357 mothers, we were able to access parity for 337. The rate attributable to primiparous was 40% (186/337) compared to 25% (83/337) in grand multiparous women (Table 2).

The major single indications for CS were obstructed labour, fetal distress, big baby, and cephalopelvic disproportion (CPD) accounting for 17.9%, 15.3%, 11.6%, and 11%, respectively. Other indications accounting for 17.6% in Table 3 included posterior occipital presentation, breech presentation, persistent occipital posterior 2% each, postdates, antepartum haemorrhage, poor uterine contraction, elective caesarean section, premature rupture of membranes, preeclampsia, congenital anomalies, placenta praevia, oblique lie, and failure of progress accounting for 1% each and the rest accounted for less than 1% and these included compound presentation, placenta abruptio, oligohydramnios, precious baby, uterine atony, eclampsia, arm prolapse, secondary arrest, cord prolapse, volvo vericose veins, and uterine rupture (Table 3).

### 3.2. Qualitative Data on the Community Perception about Caesarean Section

Qualitative data about the community perception on caesarean section delivery were collected by focus group discussions that included 6–12 individuals and each lasted for about 1–2 hours. Sixty two persons participated in the focus group discussions (FGD). The majority of the participants were males, 61.3% (38/62), while the female accounted for 38.7% (24/62). As regards to the age group, only 8.1% were teenagers, while 12.9% were above 35 years of age. Significantly, the majority had had no education or stopped in primary school, 54.8%, while only 16.1% had had tertiary education. Christians accounted for 88.7%, while Muslims made up 11.3%. Only 37.5% of the females had some form of employment compared to the 54.8% of the males (Table 4).

The majority of males, 78.9%, and females, 83.3%, largely agreed that caesarean section was a dangerous procedure. The majority of males, 76.3%, agreed that CS was for lazy women compared to 54.2% of the females. Furthermore, whereas 56.2% of the males agreed that CS leads to family breakups, only 25% of the females did agree with them (Table 5).

Recordings of the discussions were taken, and data were transcribed and summarized according to emerging themes. During FGDs, four themes emerged: the community perception of women undergoing the caesarean section, indications, advantages, and disadvantages of caesarean section delivery.

### 3.3. Theme 1: Community Perception of Women Undergoing Caesarean Section

Several myths about caesarean section emerged during the study. Caesarean section was regarded as a misfortune or curse, and in most cases, it was perceived as God's punishment to a given individual or family. It was perceived that those with physical disability had higher chances for CS compared to physically normal women. A big section of the community members also said that the use of family planning methods could be one of the reasons that women end up undergoing a caesarean section.

It was also largely perceived in Ngora that several women went for caesarean delivery in order to avoid the severe pain and vaginal tears suffered during spontaneous vaginal delivery, a finding which was mainly obtained from the male participants in the focused group discussions. This was coupled with a thought that women currently go for the operation in order to retain their vaginal canal tightness so that they maintain their sexual pleasure.

Other men had a perception that women went for CS in order to deprive them of sex since it took longer for a lady to completely heal in comparison with vaginal delivery.

One man said, “I could not even have sex regularly because she kept complaining of pain whenever I asked, so I got another woman to produce me children and satisfy me sexually.”

Some people perceived that caesarean section delivery reduces the woman's fertility and limits the number of children one can bear. A given number of people thought that caesarean section happens only if babies grow outside the uterus, and it is a way women decide to space their babies.

There is a slogan that “caesarean section is for the rich people, especially the civil servants who get a lot of money.” One man said “Those women are fat because they work in government, get a lot of money to eat, and become fat hence developing closed pelvises, and they cannot deliver normally.”

There was a perception amongst most males that CS is very common in women whose husbands are uncircumcised because such husbands are the major cause of cancer of the reproductive system which will automatically lead to caesarean section.

“Caesarean section is normally for those women with cancers down where the child passes and this is because their husbands are not circumcised,” said one 46-yr-old participant.

Some people perceived Caesarean section delivery to be due to the incompetence of the health workers, and some assume that health workers are inexperienced because some of them did not finish school so they end up failing to deliver mothers, hence resort to using theatre.

“As I was waiting for my wife while in the labour room, I heard the midwife shouting at her that; am I your mother? Did I send you to do it?” said a 28-year-old man.

#### 3.3.1. Subtheme: Doctors' Monetary Benefit

Some people perceived that women are taken for caesarean section even when the reason for carrying it out is not genuine enough but because of the doctor's personal benefit of getting money.

“It has become more of business leaving mothers with no option but to accept,” one 34-yr-old said.

#### 3.3.2. Subtheme: Dangerous Procedure

Furthermore, most mothers and males perceived CS as a risky procedure that is a matter of life and death and whose success depended on God. Someone clearly said that God knows it all. According to the people in Ngora, CS is associated with a lot of death due to the various intraoperative and postoperative complications involved and also bad effects of the anaesthetic drugs.

One young man sadly reported that “my elder sister died one day after her caesarean section and the doctor told us that she had lost a lot of blood during the operation.”

#### 3.3.3. Subtheme: Laziness

There was a belief that CS is for lazy women who could not produce normally.

“Laziness of some mothers also contributes to CS deliveries in Ngora district as some of them feel they cannot push the baby through the vaginal canal and therefore opt for CS since it just involves cutting the stomach to remove the baby,” one elderly man said.

“Those operations are for these soft girls, ones who did not eat atapa (local name for millet bread),” said a 37-year-old woman.

### 3.4. Theme 2: Perceived Indications and Risk Factors for Caesarean Section Delivery

#### 3.4.1. Subtheme: Previous Scars

People in Ngora attributed CS to previous caesarean section scars, too much pain from the previous SVD, and mothers whose babies used to die during birth; in order to save the precious baby, CS is the only choice. There is a case of a mother who said, “My children used to die during delivery, so I decided to go for an operation, and the baby was picked out alive. I was really so happy.”

#### 3.4.2. Subtheme: Medical Deformities

Some people reported that some women have natural maternal deformities that are indications for CS to be carried out like sharp pelvic bones, narrow waist, and contracted pelvises which may arrest the baby during labour.

“I see every disabled woman who gets pregnant gets operated, I think they all have a narrow opening,” said a 27-year-old man.

“Women with disabilities!… paused a 28-year-old woman, what do they look for in men? They can deliver. They have to be operated and have no good care thereafter,” a 28-year-old woman wondered.

Disabilities due to fractures of the hip bones and intramuscular injections were also reported by people in Ngora as some of the other indications of mothers to opt CS.

“These injections have caused disability, women now have narrow birth canal, if we reduced them we can reduce caesarean sections,” said one 26-year-old male.

“Diseases such as pressure and asthma have become very common nowadays in women. These women have to be operated,” said one 29-year-old lady.

People in the Ngora district also contributed to indications of caesarean section such as fetal distress, uterine rupture, and long lasting labour pains.

#### 3.4.3. Subtheme: Maternal Age

Some people argued that the rising teenage pregnancies in Ngora may be a risk factor to CS especially the primigravidas in whom young girls with underdeveloped bodies attempt to carry pregnancies with the narrow birth canals resulting in failure to deliver vaginally.

“These young irate girls are a problem, you find a 16-year-old pregnant, what do you expect! she can't push a baby, exclaimed one man.

People in the Ngora district also shared the fact that mothers conceiving at above 40 years and multiparous mothers will definitely undergo CS. In addition, lack of physical exercise, stress during pregnancy, failure to follow health workers' advise, and also ill health of the mothers, for example, hypertension, asthma, and poor nutrition during pregnancy, are also risk factors to CS in the Ngora district.

### 3.5. Theme 3: Perceived Advantages of Caesarean Section Delivery

#### 3.5.1. Subtheme: Lifesaving

It is also thought that some women in the Ngora district have priority for the traditional birth attendants because they are cheap. This is said to be a risk factor since they may fail to deliver the mothers with malpresentation of the baby, placental prolapse, nuchal cord, and big baby, thus by the time she is referred to the hospital, CS is the only lifesaving procedure.

Most of the people said that CS is a lifesaving process because it saves both the mother and the baby in case vaginal delivery fails and that also relieves pain that the mother gets when labour fails to progress for the baby to come out.

A 27-year-old woman said that “for me it saved my life and that of my baby and we are now living happily.”

#### 3.5.2. Subtheme: Prevents Mother-to-Child Transmission of HIV

Importantly, CS prevents mother-to-child transmission of HIV since there is careful carrying out of the baby from the womb and cutting of the cord.

One mother in her 30 yrs said, “It must be a way of saving children from getting HIV since the child is gotten out carefully and cord cut.”

### 3.6. Theme 4: Perceived Disadvantages of Caesarean Section Delivery

#### 3.6.1. Subtheme: Expensive Procedure

Majority of the people said that CS is an extremely expensive procedure.

One man even said, “I would even escape because of the costs.”

#### 3.6.2. Subtheme: Family Breakups

CS has led to some of the family breakups in Ngora since the man's relatives take it as a curse to the family, and the man is denied sexual intercourse by the woman due to too much pain which result in the man looking for another woman to satisfy him sexually.

“I would get another woman to produce for me children normally and also satisfy me sexually,” echoed one 24-year-old man.

“Women who undergo CS take long to heal …pause.., it reduces the woman's physical productivity,” said one lady.

“Laziness of some mothers also contributes to CS deliveries in Ngora district as some of them feel they cannot push the baby through the vaginal canal and therefore opt for CS since it just involves cutting the stomach to remove the baby” one elderly man said.

“Some women become a laughing stock because of delivering by CS as the other women look at her as one who cannot push a baby normally and can only bear a child after being operated,” said another.

## 4. Discussion

Of the 2573 mothers, 14% (357/2573) were delivered by CS. This is a facility-based CS rate at the lowest point in Uganda at which comprehensive emergency obstetric care (CEmOC) services are provided. This range is higher than the range of 4–8% in an earlier Ugandan study in 2016 [16].

Significantly, this study shows that the rate of caesarean section is rising. A study done in Uganda by extraction of Uganda National Health Management Information System (HMIS) reports between 2012 and 2016 highlighted an overall increase in facility-based CS from 19% in 2012 to 22% in 2016. Furthermore, there was a variation of the rates at different CEmOC centers with the highest CS rates occurring in general hospitals (22–32%), followed by referral hospitals (20–25%) and Health Centre IV facilities (4.0–8.0%), with Health Center IV level having the highest annual increase in CS rates, followed by general hospitals. This study shows a rate higher than the average as per Emily et al.'s study. [16]. Furthermore, the rate is higher than that found amongst lower health facilities which stood at 8–9%, yet closer to that of higher-level facilities that was in the range of 16–18% in a study done in the Kabarole district of western Uganda. Moreover geographical differences may contribute to the variations.Kabarole is located in the western part of the country, while Ngora is located in the east. In the Uganda health care system, Ngora facilities rank as the lowest health facilities at which CEmOC can be provided. These differences and similarities could be explained by the conclusions made in the 2012 to 2016 HMIS data analysis that found Health Centre IVs being responsible for the sharp rise of caesarean section rates and regional heterogeneity in caesarean section rates [16]. The study by Emily et al. so further projected a CS rate rise of 36% by 2021. However, with Ngora facilities at 14%, this is a 75% rise from a maximum rate of 8% to 14% for Health centre IVs [16].

However, our study was in agreement with a study in Pakistan, where it was revealed the percentage of CS was 13.6% [24]. Worth noting is that the Pakistan study was a cross-sectional review of the Pakistan Demographic Health Survey data rather than a facility-based one. Moreover, Uganda, like Pakistan, is a developing country, and the decision to do a caesarean section is largely a decision of the medical profession rather than on demand by the mother [24, 25]. Also, the high rate could be attributable largely to a lack of operative vaginal delivery services and probably lack of very critical staff like obstetricians. Research has shown that simple algorithms with timely tutorials can help junior staff to improve their decision-making processes where the preferable alternative of continual senior support is not feasible, and this can significantly reduce caesarean section rates [26].

Our study found a rate lower than that found in Addis Ababa teenagers, Ethiopia, that reported a rate of 19.2% and 18% reported at St. Joseph Medical Hospital in Tanzania [27, 28]. However, the study in Ethiopia focused only on teenagers and was done in an urban setting where studies have demonstrated a likelihood of higher prevalence compared to the rural settings, but also in both studies, the facilities are at a higher level than Ngora facilities [25, 27].

Caesarean section rates tend to show heterogeneity with rates ranging from as low as 5.5% in the poorest women to as high as 35.77% in the richest women in cities [11]. Studies have shown that the more educated, economically empowered, and urban dwelling women have a more likelihood of having CS. This is because they are likely to attend a private facility in which the procedure may be done not only for medical indications but also on demand [24, 25, 29, 30].

This may by extrapolation of Uganda settings mean that the CS rates in higher-level facilities and urban base facilities are likely to be higher than that of Ngora facilities that are rural based and being at the bottom level of CEmOC service providing facilities. No wonder the study by Emily et al. demonstrated levels above 20% for referral facilities compared to 4–8% in Health Centre IVs [16].

### 4.1. Indications for Caesarean Section

Like the Uganda survey showed heterogeneity in the caesarean section rate, there is heterogeneity in indications [16]. Private facilities that are mostly located in the urban areas that see the privileged class of women seem to have a rate higher than the rural public facilities [20, 24, 25, 29].

In our study, the top three indications for caesarean section were obstructed labour 18%, fetal distress 15%, and big baby 12%. In a Bangladesh study, the authors reported that the major clinical indications for CS were previous CS (35%), prolonged and obstructed labour 15%, fetal distress 11%, and amniotic fluid disorder 11% [30]. In another study in Iraq, the main overall indications for caesarean section were a previous caesarean section (70.49%), cephalopelvic disproportion (35.31%), and mother's request 14.26% [11]. In a study done in Tanzania, prolonged or obstructed labour accounted for 30%, malpresentation of the baby 20%, and fetal distress 11% [28]. Another study done in Muhimbili National referral hospital in Tanzania ranked the top three indications for CS as repeat CS (30.2%), obstructed labour (14.4%), and fetal distress (13.6%) [31].

In a study conducted in a tertiary hospital, Fort Portal Regional Referral Hospital (FRRH) in western Uganda, the most common group of indications for caesarean was dystocia 43.5%, followed by fetal distress 18.5%, previous scar 17%, malpresentation 10.5%, and maternal compromise 10% [32]. The differences could be attributed to the level of the health facility, and thus presence of senior staff like obstetricians. This could in turn have influence on the cases received at the facility. For example, the FRRH is a referral centre for various districts and more likely to get mothers with complications or ones that have been trying labour from elsewhere compared to the lower centers in Ngora. But, in agreement with our study is that overall obstructed labour was still the leading indication of caesarean section.

The issue of previous scars has been raised as responsible for the increasing rate of CS, and the majority of mothers who present with a previous scar are likely to end up with a repeat caesarean section [33]. In the Ngora district, it accounted for 9.3%. In FRRH, it accounted for 17%, while it made up 35% of the cases in Bangladesh [13, 17]. This could be attributed to the presence of specialists in the latter two areas, and thus high-risk mothers such as with previous scars are likely to seek care from there.

In contrast to a study in Iraq, we do not have a significant number of women who will have CS on request. This could be because this study was in a rural area with low social economic and less-educated mothers. Career pursuit is likely to delay parturition in mothers, and older primigravidas are more likely to deliver by CS compared to their young counterparts. A mother who has had an index CS is likely to end up with a consequent one compared to one without. This could explain the differences in the rates of repeat CS in the Iraq study when compared to ours [11, 24, 25, 34].

### 4.2. Qualitative Data Discussion

According to the people in Ngora, CS is associated with a lot of deaths due to the various intraoperative and postoperative complications. This agrees with the study in Ghana in which slightly above 50% of the expectant mothers interviewed considered it to be dangerous to the mother and the baby, and 6% indicated they would refuse it even if when it was medically indicated [21].

In a study done in Ghana to assess awareness, perceptions, and attitudes towards caesarean section amongst antenatal care (ANC) attendees who had never had any previous surgical operation, 93.3% preferred vaginal delivery against the 3.5% for caesarean section. The majority said that vaginal delivery was the natural way to deliver (64.7%), safer (19.2%), cheaper (4.7%), reduced postpartum morbidity (11.4%), and resulted in early hospital discharge. The 3.5% who preferred CS did so because they wanted to avoid the labour pains [21]. Like in the study above, most women preferred vaginal delivery (VD) since CS was very risky whose success depended on the will of God. This finding is also in agreement with studies done outside Africa. A study in Chile revealed 91.5% of interviewed women in antenatal preferring to have a vaginal delivery [35]. In a study involving 180 mothers in Chile, 77.8% of the women preferred vaginal delivery, while in another carried out in Australia amongst 290 gravid mothers, 93.5% preferred VD [36, 37].

However, like in a study in Ghana, the population seemed to be positive about genuine indications for caesarean section. In this study, although 40% perceived that most women undergoing CS may die, 95.7% were willing to undergo the operation when indicated. However, 4.3% of the pregnant women would refuse the surgery even if indicated [38]. Other participants in our FGDs supported it especially as not the only way to minimize the aura of pain from the previous VD or a previous C-section but also as a safer way to have a live baby, following previous still birth. These perceptions are echoed in a study by Tahmina Begum et al. in Bangladesh.

“Before my case, none of my family members ever attended hospital for birthing purpose. As a part of the tradition, I also went to my natal home and they called our family ‘daima' [traditional birth attendant]. When labour pain started, my baby defecated inside the uterus and instantly she referred me to this hospital. Consequently, caesarean delivery saved my baby's life” (postcaesarean mother) [22].

“I prefer caesarean delivery. I heard baby delivered by caesarean section had a healthier brain compared to normally delivered baby as it does not stay in the birth canal for a long time” (quote from a pregnant woman) [22].

Men largely agreed that CS was a misfortune. They described it as a curse and God's punishment to the woman and the family. There is a paucity of the literature regarding this male-held view. Women and men alike voiced their negative voices regarding CS as a mode of delivery. They preferred vaginal delivery as it is natural and described caesarean section as being expensive, a curse, and an indictment on incompetence of health workers. Caesarean section was highlighted as denying sex to men. It was viewed as being for lazy women. It was held responsible for family breakups. These findings are echoed in another study in Uganda by Dusabe et al. in which women that underwent CS were considered lazy [17]. The findings could shade light on why although 82% of the women said they would accept a C-section in a study in Nigeria, they would do so if the husband consented despite their disapproval because he is the head of the family and therefore wants the best for the mother and the baby [39].

The inclination by women to vaginal delivery as a natural way and one associated with less recovery time and ability to go on with usual chores and responsibilities has also been highlighted by studies in Brazil, Bangladesh, and Ghana [22, 23, 38]. Both pregnant and postcaesarean women in the Bangladesh study expressed their preferences for vaginal birth irrespective of the cost of caesarean section. The reasons for vaginal delivery preferences raised included faster postpartum recovery and living in extended families where elderly members who were the main decision makers had a strong preference for vaginal birth. In a study in Bangladesh, a twenty five-year-old pregnant woman, during an FGD, commented on why having a caesarean was difficult and painful.

“We need to carry paddy [rice] bundle over our head and need to feed our cattle. If suddenly rain comes we have to take the cut [rice] paddy from the yard to inside the house. In that case if I would have caesarean section, I will not be able to run. Women's lives become really handicapped after having this operation” (quote from a pregnant woman) (Tahmina Begum, 2018).

Further still, although considered painful, vaginal delivery was preferred because probably pain was considered as a sign of endurance and one required little time to heal. Other studies have echoed this finding as most subjects considered labour pain as a natural and necessary part of pregnancy and cheap [35, 40, 41].

It was largely echoed that CS was a very expensive procedure. This could be explained by out-of pocket payments at these facilities. Although government of Uganda policy is to offer free maternal services at the government facilities, out-of-pocket payments due to frequent stock-outs and unavailability have been proven to push the patients into deeper economic hardships in Uganda [42] and elsewhere [43] and to curtail accessibility to services. Our finding of the peoples' lamentation is similar to findings that indicated that government funding truly improved utilization of CS services in low-income countries.

### 4.3. Limitations

Some indications were not clear such as nuchal cord, multiple pregnancy, CPD, and big baby. Since it was retrospective data, we were not able to objectively tease out what the indication clearly was.

## 5. Conclusions

This study demonstrates a 75% rise in the caesarean section rate in Health Centre IVs in Uganda. This is more than double the earlier anticipated rise of 36%. The WHO population-based studies have indicated that CS rates of 5% could achieve major improvement on maternal outcomes and better neonatal health; rates between 5% and 10% have been reported to attain better outcomes. There is a need to have a facility level-based study to determine the rate of CS that is lifesaving or at what rate do additional CS shows no benefit in curtailing maternal and neonatal mortality and morbidity. Studies do not show added benefit of rates greater than 15%.

The indications need to be made clearer and explained to the clients and their spouses (if present) and preferably by the doctors who do the operations. This will help the couples to understand the indications better and dispel the unnecessary fears and myths such as CS being a curse, being carried out to deny men sex, and only for the lazy women.

## Figures and Tables

**Table 1:** Number and mode of delivery of women and their age groups.

Month/year	VD (%)	CS (%)	Total deliveries			Percentage distribution of caesarean section rate by age in years (%)		
						15–19	20–24	≥25
April 2018	166 (93)	12 (7)	178			1 (8.3)	2 (16.6)	9 (75)
May 2018	205 (87)	31 (13)	236			9 (29)	7 (22.6)	15 (48.4)
June 2018	180 (82)	40 (18)	220			12 (30)	15 (37.5)	13 (32.5)
July 2018	170 (87)	25 (13)	195			11 (44)	8 (32)	6 (24)
August 2018	200 (89)	25 (11)	225			6 (24)	7 (28)	12 (48)
September 2018	204 (88)	27 (12)	231			7 (25.9)	6 (22.2)	14 (51.9)
October 2018	196 (84)	36 (16)	232			11 (30.6)	9 (25)	16 (44.4)
November 2018	96 (73)	36 (27)	132			6 (16.7)	15 (41.7)	15 (41.7)
December 2018	258 (87)	39 (13)	297			6 (15.4)	12 (30.8)	21 (53.8)
January 2019	213 (90)	24 (10)	237			10 (41.70)	8 (33.3)	6 (25)
February 2019	173 (84)	33 (16)	206			9 (27.3)	11 (33.3)	13 (39.4)
March 2019	155 (84)	29 (16)	184			9 (31.0)	13 (44.8)	7 (24.1)
Total	2216 (86)	357 (14)	2573			97 (27.2)	113 (31.7)	147 (41.2)

Total number of mothers in each age category (%)			15–19	20–24	≥25			
			618 (24)	926 (36)	1029 (40)			

**Table 2:** Percentage of caesarean section delivery according to parities of mothers.

Gravidity	Number of caesarean sections	Percentage of caesarean sections
Primiparous	136	40
Multiparous	118	35
Grand multiparous	83	25
Total	337	100

**Table 3:** Summary of indications for caesarean section delivery in the Ngora district.

Indication	Fetal distress	Obstructed labour	Big baby	Cephalopelvic disproportion (CPD)	Previous scar	Prolonged labour	Cervical dystocia	Multiple pregnancy	Nuchal cord	Transverse arrest	Others
Number	71	83	54	51	43	20	19	19	12	10	82
(%)	15.3	17.9	11.6	11	9.3	4.3	4.1	4.1	2.6	2.2	17.6

**Table 4:** Characteristics of the focus group discussion participants.

Sex	24 females (38.7%)	38 males (61.3%)	Total numbers (male + female = 62)
Variable	Number (%)	Number (%)	Number (%)
*Age*			
≤19	3 (12.5)	2 (5.3)	5 (8.1)
20–24	8 (33.3)	10 (26.3)	18 (29)
25–29	7 (29.2)	12 (31.6)	19 (30.6)
30–35	3 (12.5)	9 (23.7)	12 (19.4)
>35	3 (12.5)	5 (13.2)	8 (12.9)

*Education level*			
No formal education/primary	13 (54.2)	21 (55.3)	34 (54.8)
Secondary	8 (33.3)	10 (26.3)	18 (29.0)
Tertiary	3 (12.5)	7 (18.4)	10 (16.1)

*Religion*			
Catholic	10 (41.7)	14 (38.9)	24 (38.7)
Anglican	6 (25)	10 (27.8)	16 (25.8)
Pentacostal	6 (25)	7 (19.4)	13 (21)
Seventh day Adventist (SDA)	1 (4.2)	0 (0)	1 (1.6)
Muslim	1 (4.2)	6 (16.7)	7 (11.3)

*Occupation*			
Formal	7 (29.2)	8 (21.1)	15 (24.2)
Informal	2 (8.3)	17 (44.7)	19 (30.6)
Unemployed	15 (62.5)	13 (34.2)	28 (45.2)

**Table 5:** Generated themes and myths generated from the focus group discussion.

Themes and subthemes generated from focus group discussion	Number, *n*, and percentage (%) of respondents					
	Male			Female respondents		
	Yes	No	Undecided	Yes	No	Undecided
*Theme 1: community perception of women undergoing caesarean section*						
Subtheme: doctors' monetary benefit	25 (65.8)	5 (13.2)	8 (21.1)	12 (50)	8 (33.3)	4 (16.7)
Subtheme: dangerous procedure	30 (78.9)	5 (13.2)	3 (7.9)	20 (83.3)	2 (8.3)	2 (8.3)
Subtheme: laziness	29 (76.3)	7 (18.4)	2 (5.3)	10 (41.7)	11 (45.8)	3 (12.5)

*Theme 2: perceived indications and risk factors for caesarean section delivery*						
Subtheme: previous scars (from previous caesarean section and vaginal delivery)	20 (52.6)	10 (26.3)	8 (21.1)	17 (70.8)	2 (8.3)	5 (20.8)
Subtheme: medical deformities	16 (42.1)	4 (10.5)	18 (47.4)	20 (83.3)	4 (16.7)	0 (0)
Subtheme: maternal age	18 (47.4)	15 (39.5)	5 (13.2)	19 (79.2)	3 (12.5)	2 (8.3)

*Theme 3: perceived advantages of caesarean section delivery*						
Subtheme: lifesaving	15 (39.5)	16 (42.1)	7 (18.4)	20 (83.3)	2 (8.3)	2 (8.3)
Subtheme: prevents mother-to-child transmission of HIV	21 (55.3)	5 (13.2)	12 (31.6)	17 (70.8)	5 (20.8)	2 (8.3)

*Theme 4: perceived disadvantages of caesarean section delivery*						
Subtheme: expensive procedure	34 (89.5)	4 (10.5)	0 (0)	20 (83.3)	4 (16.7)	0 (0)
Subtheme: family breakups	20 (52.6)	15 (39.5)	3 (7.9)	6 (25)	17 (70.8)	1 (4.2)

## Data Availability

The database is available on request, contact e-mail: Jntezi@gmail.com.

## Abbreviations

ANC: Antenatal care
CEmOC: Comprehensive emergency obstetric care
COBERS: Community Based Education and Research Services
CS: Caesarean section
CPD: Cephalopelvic disproportion
HMIS: Health Management Information System
FGD: Focus group discussion
FRRH: Fort Portal Regional Referral Hospital
MDG: Millennium Development Goals
MMR: Maternal mortality ratio
SDGs: Sustainable Development Goals
VD: Vaginal delivery
WHO: World Health Organization.

## Additional Points

*Strength*. This study was able to emphasize the role of male involvement in decision-making and why medical staff need them during counseling and consenting for CS. It further highlights the wide-spread misconceptions about CS such as being for the lazy women and a mischief.

## Supplementary Materials

Supplementary Materials Focus group guide used during the qualitative data collection.
