# Supplementary material for: Prevalence, Indications, and Community Perceptions of Caesarean Section Delivery in Ngora District, Eastern Uganda: Mixed Method Study
Source: Obstet Gynecol Int. 2020 Jul 20;2020:5036260. doi: 10.1155/2020/5036260 (PMC7387994; doi:10.1155/2020/5036260)
Supplement: Supplementary Materials — Focus group guide used during the qualitative data collection. [file 5036260.f1.pdf]

GUIDE QUESTIONS

These are the guide questions that were used for the Ngora research titled ‘PREVALENCE, INDICATIONS AND COMMUNITY PERCEPTIONS OF CAESAREAN SECTION DELIVERY IN NGORA DISTRICT EASTERN UGANDA: MIXED METHODS STUDY’

General guiding questions

1. Tell us your experiences on c/section delivery and how you would feel if you were told that someone close to you was going for c/section
2. Some of you people said that women can die in theatre, what do you think makes women to die in theatre?
3. In other places very many women deliver by c/section how common is c/section in this community?
4. Can we explore the reasons to why women cannot deliver normally and they are taken for c/section
5. We also want to know from you if c/section has its benefits?
6. Are there cases of women who request for c/section in your community?
7. What could be the reasons as to why they ask for c/section?

END

FOCUS GROUP DISCUSSION TO DETERMINE THE COMMUNITY PERCEPTION ON CAESAREAN SECTION DELIVERY CONDUCTED IN AGOLITOM VILLAGE

Q1. Let us talk about cesarean section. What is it?

Q2. How would you feel if any of your relatives or yourself was to go for a cesarean section?

Q3. How common is Cesarean section in this community?

Q4. Does this form of delivery bear any benefit?

Q5. Share with us what you think are the advantages of this form of delivery

Q6.And what do you think are the disadvantages of cesarean section?

Q7.What are some of the reasons for cesarean section?

Q8. Are there some mothers in this community who willingly go in for cesarean section?

Q9. Why do some mothers willingly opt for cesarean section?

END

FOCUS GROUP DISCUSSION TO DETERMINE THE COMMUNITY PERCEPTION ON CESAREAN SECTION CONDUCTED IN OKOROM VILLAGE

Participants: males

1. Tell us your experiences about c/section and how you would feel if told you, your sister, mother or wife was going for c/s?
2. Most of you seem worried of whether the woman will come back alive or not, what do you think can make woman to die during or after c/section?
3. How common is c/s in your community?
4. What could be leading to many women delivering c/section in this village?
5. Do you think c/section has some advantages?
6. Since we have agreed that it has some benefits, can we share the benefits you know?
7. Tell us the disadvantages of c/s that you know
8. In some places where we have been, some women walk to hospital and request for c/section. Are such people also in this village?
9. What could make other people to request for c/s?

END

FOCUS GROUP DISCUSSION TO DETERMINE THE COMMUNITY PERCEPTION ON CESAREAN SECTION DELIVERY

Participants: females attending to women at the facility (maternity and postnatal)

1. What is your experience about C-Section and how would you feel if your relative is going to be taken to theater for C-section.What do the rest of you think?
2. How common do you think C-section is in this community?
3. What do you people think are the reasons women in this community end up in C-section.
4. In your opinion, how do you think C-section has helped the community and the mothers?
5. What do you think are the disadvantages of C-section in your opinions?So what do the rest of you think?
6. Are there people who request for C-section in your community? What would you comment about women who go for C/Section willingly
   Probe; Oh what kind of people are these and what are their reasons.
   Experience 4

Probe; was this your first pregnancy? Tell me about the pain.

7. Tell me about the reasons people deliver by C-section. What do the others think?
8. Which kind of women in this community have high chances of going for C-section?

9. To the other family members are there any effects, if yes tell me about them.What do the rest think?
10. How common are cesarean section deliveries in this community?
11. What about you number 4 what do you think

12. What is your comment on women who walk to theatre to get cesarean section willingly?

13. Compare delivery by cesarean section and normal delivery, and why do you think so?

END

FOCUS GROUP DISCUSSION CONDUCTED IN KOBUKU VILLAGE TO DETERMINE THE COMMUNITY PERCEPTION ON C/SECTION

Participants: males attending to women in the maternity and postnatal wards

1. Tell us about the reasons people deliver by C-section
2. Which kind of women in this community have high chances of going for C-section
3. How the people in the community comment about cesarean section
4. To the other family members are there any effects, if yes tell me about them
5. What do the rest think?
6. How common are cesarean section deliveries in this community?
7. What about you number 4 what do you think
8. What is your comment on women who walk to theatre to get cesarean section willingly?
9. Compare delivery by cesarean section and normal delivery, and why do you think so?

END

FOCUS GROUP DISCUSSION CONDUCTED IN APAMA VILLAGE TO DETERMINE THE COMMUNITY PERCEPTION ON CESAREAN DELIVER

Participants: male

1. Cesarean section is a form of delivery that some mothers undergo; tell me how you would feel if your wife, sister or any of your relative is to be operated
2. I believe you know some mothers that have had C sections, it might have been your mother, sister, or a neighbor so on your observation how common do you think is C section delivery common in this community?

3. OK, so you all said that C section delivery is very common in this community and someone would wonder what would be the ultimate issue for the high numbers, please tell me in your opinion what could be the reasons as to why these mothers end up in theater for cesarean sections.
4. Anything you would wish to add on that?
5. Do you think this form of delivery has advantages?
6. Tell me some of the advantages C section has and why you would think so
7. Share with me the probable disadvantages of C section as another form of delivery
8. We have been talking about cesarean section where someone must be operated as a lifesaving procedure but are there mothers who just go to the hospital and ask for the cesarean section, if yes, tell me what you think are the reasons for choosing C section delivery

END

FOCUS GROUP DISCUSSION CONDUCTED IN APAMA VILLAGE TO DETERMINE THE COMMUNITY PERCEPTION ON CESAREAN DELIVER

Participants: females of child bearing age

1. Tell us you experience about c/section and how you would feel if you were told that someone you know is going for c/section?
2. What do you think leads to women undergoing c/section
3. Does c/section have any benefits
4. Thank you for your responses, Can we now share those few benefits in detail
5. What do you think makes some people to go and request for c/section
